# Short-term tissue decomposition alters stable isotope values and C:N ratio, but does not change relationships between lipid content, C:N ratio, and Δδ^13^C in marine animals

**DOI:** 10.1371/journal.pone.0199680

**Published:** 2018-07-18

**Authors:** Matthew J. Perkins, Yanny K. Y. Mak, Lily S. R. Tao, Archer T. L. Wong, Jason K. C. Yau, David M. Baker, Kenneth M. Y. Leung

**Affiliations:** The Swire Institute of Marine Science & School of Biological Sciences, The University of Hong Kong, Pokfulam, Hong Kong, China; Universidad de la Republica Uruguay, URUGUAY

## Abstract

Measures (e.g. δ^15^N, δ^13^C, %C, %N and C:N) derived from animal tissues are commonly used to estimate diets and trophic interactions. Since tissue samples are often exposed to air or kept chilled in ice over a short-term during sample preparation, they may degrade. Herein, we hypothesize that tissue decomposition will cause changes in these measures. In this study, we kept marine fish, crustacean and mollusc tissues in air or ice over 120 h (5 days). We found that tissue decomposition in air enriched *δ*^15^N (range 0.6‰ to 1.3‰) and *δ*^13^C (0.2‰ to 0.4‰), decreased %N (0.47 to 3.43 percentage points from staring values of ~13%) and %C (4.53 to 8.29 percentage points from starting values of ~43%), and subsequently increased C:N ratio (0.14 to 0.75). In air, while such changes to *δ*^13^C were relatively minor and therefore likely tolerable, changes in *δ*^15^N, %N, %C and C:N ratio should be interpreted with caution. Ice effectively reduced the extent to which decomposition enriched *δ*^15^N (≤ 0.4‰) and *δ*^13^C (≤ 0.2‰), and eliminated decomposition in C:N ratio, %N and %C. In our second experiment, for fish tissues in either air or ice over 120 h, we observed no effects of decomposition on relationships between lipid content, C:N ratio, and *Δδ*^13^C (change in *δ*^13^C after lipid removal), which are employed to correct *δ*^13^C for samples containing lipid. We also confirmed that lipid in tissues caused large errors when estimating *δ*^13^C (mean ± standard error = -1.8‰ ± 0.1‰, range -0.6‰ to -3.8‰), and showed both lipid extraction and mathematical correction performed equally well to correct for lipids when estimating *δ*^13^C. We, therefore, recommend that specimens of marine animals should be kept in ice during sample preparation for a short-term, as it is an effective means for minimizing changes of the stable isotope measures in their tissue.

## Introduction

Stable isotope ratios of nitrogen (^15^N/^14^N relative to atmospheric nitrogen, termed *δ*^15^N) and carbon (^13^C/^12^C relative to Vienna PeeDee Belemnite, termed *δ*^13^C) are widely used within aquatic and terrestrial ecological research for dietary reconstruction of consumers [1] and determining trophic structure of communities [2–4]. Derived ecological conclusions are dependent upon the accuracy of *δ*^15^N and *δ*^13^C estimates. Research has accordingly investigated potential sources of error when estimating *δ*^15^N and *δ*^13^C, including error associated with methods of sample preservation [5–8] and sample preparation [9,10]. Studies have shown that in some instances, such error is significant. For instance, Tarroux et al. [11] showed that the presence of lipid within tissues affected estimates of *δ*^13^C and subsequently biased estimates of resource contributions to consumers when using dietary mixing models. Post [12] showed the use of inappropriate *δ*^15^N discrimination values (the change in *δ*^15^N from resource to consumer) introduced error into trophic structure estimates of food chain length, while Perkins et al. [10] proved that selection of different tissue types during sample preparation could lead to such inappropriate *δ*^15^N discrimination values. Therefore, error in estimates of *δ*^15^N and *δ*^13^C introduced during sample preservation and preparation in many instances must be avoided or accounted for, though this is easily achieved once such error has been documented and is widely understood. As chemical preservation [13,14] and in a limited number of instances even freezing [7,15,16] may confound the fidelity of stable isotope values, many studies opt to keep samples unpreserved or in/on ice until advanced sample preparation is undertaken. Currently, however, we have limited empirical evidence to assess if and how tissue decomposition may affect *δ*^15^N and *δ*^13^C within unpreserved animal tissues in the short term (e.g. < 1 week).

Decomposition of samples has been better investigated for plants and algae, showing complex and variable effects [17,18]. Conversely, studies on animals have been of restricted scope. Payo-Payo et al. [19] and Burrows et al. [20] opportunistically sampled stranded cetaceans and turtles (*n* = 1 to 3) of unknown time of death, observing little change in either *δ*^15^N and *δ*^13^C over 62 or 14 days, respectively. Scarce studies employing greater experimental control have shown mixed results. Observations of a terrestrial invertebrate (*Drosophila*; [13]) and marine vertebrates (seal, shark, trout; [21]) have shown either no change or limited change (*δ*^15^N enriched by 0.4‰; *δ*^13^C depleted by 0.4‰ to 0.8‰) over a short-term (≤ 10 days). Variability amongst results and differences in experimental conditions across studies necessitates that further research is needed to determine if there exists consistent and generalisable patterns in how tissue decomposition affects stable isotope measures, or if decomposition effects are largely species- or tissue-specific. Given that tissues undergo both biological breakdown from microorganisms and self-breakdown with cessation of metabolism following death, we expect the chemical composition of tissue samples to change, either through loss of their constituents, change in relative proportion of different constituents, or formation of new constituents through cellular breakdown or deposited microorganism residues [18, 22, 23]. Thus, empirical evidence is required to inform researchers about the extent to which tissue decomposition may alter *δ*^15^N and *δ*^13^C in the short-term, for a broader range of species using fresh specimens.

In addition to *δ*^15^N and *δ*^13^C, ratios of percentage carbon (%C) to nitrogen (%N) (termed C:N ratio) of animal tissues provide a proxy of lipid to protein content and so can be informative of both lipid content and diet [24,25]. To date, empirical studies have barely examined potential tissue decomposition effects on C:N ratios of tissues following sampling (but see [18,21]). Decomposition is likely to proceed by physical breakdown and loss of tissues’ structural components, including nitrogen and carbon, so any decomposition would likely infer a change in C:N ratio (unless %C and %N degrade at rates that maintain this ratio). However, it is still unclear how tissue losses of nitrogen and carbon correspond to the heavier or lighter isotopes of each element. Losses in %N and %C may be associated with enriched *δ*^15^N and *δ*^13^C, respectively, provided that microbial or self-breakdown processes preferentially involve liberation of lighter metabolically more-active ^14^N and ^12^C from tissue, respectively [21].

Importantly, C:N ratios are also widely used to provide an *a posteriori* mathematical correction for *δ*^13^C values [26]. This is because tissues used for stable isotope analysis (SIA) often contain lipid, which is naturally depleted in *δ*^13^C [27]. This is a problem as lipid content varies between species, individuals, and tissues within individuals, and thus disproportionately affects estimates of *δ*^13^C in some samples, so needs to be accounted for [10]. However, lipid extraction prior to SIA can be costly and laborious, and may affect *δ*^15^N values [9,11,26]. Fortunately, the removal of lipids in a subset of samples can allow for the use of a derived equation of the relationship between C:N ratio and *Δδ*^13^C (the change in *δ*^13^C following removal of lipids) to provide mathematical correction of remaining samples simply based on their C:N ratios. The relationship between C:N ratio and *Δδ*^13^C follows two other important relationships between lipid content (%) and *Δδ*^13^C, and between C:N ratio and lipid content. The mathematical correction is extensively used, with a main paper reporting this procedure [26] currently cited >1000 times. Therefore, it is desirable to understand if and how tissue decomposition may affect the application of this mathematical correction. Decomposition may ultimately affect estimates of *δ*^13^C in two ways; either through using degraded tissues to derive the correction equation itself, or through using ‘fresh’ tissues to derive the equation but its subsequent application to correct *δ*^13^C for degraded tissue samples. In either instance, the use of a mathematical correction to estimate *δ*^13^C maybe robust to tissue decomposition unless decomposition, which includes microbial processes, adds or depletes compounds at a sufficiently high mass to alter relationships between C:N ratio, *Δδ*^13^C and lipid content of a tissue sample (i.e. tissue C:N ratio not matched by lipid content). In many instances, other approaches are used to estimate *δ*^13^C, including SIA only (where no attempt is made to correct / control for lipids in tissues), or lipid extraction prior to SIA. It would therefore be insightful to contrast how tissue decomposition may differentially affect estimates of *δ*^13^C made through either SIA only, lipid extraction prior to SIA, or mathematical correction after SIA.

In this study, we investigated the potential effects of tissue decomposition on multiple tissue measures in selected marine organisms over time. We conducted two separate experiments to answer three questions. *Experiment 1* –*Q1*. How does tissue decomposition affect *δ*^15^N, *δ*^13^C, %N, %C and C:N ratio? *Experiment 2* –*Q2*. Does tissue decomposition affect tissue lipid content or relationships between (i) lipid content and *Δδ*^13^C, (ii) C:N ratio and lipid content, and (iii) C:N ratio and *Δδ*^13^C? and *Q3*. Are alternative methodologies for estimating *δ*^13^C (SIA; lipid extraction then SIA; SIA then mathematical correction) differentially affected by tissue decomposition?

## Materials and methods

### Ethics statement

Freshly killed fishes, crustaceans and molluscs were brought from Aberdeen wholesale fish market, Hong Kong (coordinates: 22.248020, 114.150744). We were able to easily observe animals being killed in the market (i.e. removed from their tanks and left to expire), and so purchased animals for which we knew precise time of death. All individuals were inspected to ensure no visible damage or signs of poor health.

### Overview

Here we describe two separate experiments. The first experiment provides a simple test of how decomposition affects common tissue measures. The second experiment tests if decomposition affects a widely applied methodological procedure that is used *a posteriori* to correct *δ*^13^C values against error caused by lipid content.

### Experiment 1 ― *Q1*. How does tissue decomposition affect *δ*^15^N, *δ*^13^C, %N, %C and C:N ratio?

#### Sample preparation

Individuals of two fish species (grouper *Cephalopholis boenak*; rabbitfish *Siganus canaliculatus*), two crustacean species (mantis shrimp *Miyakea nepa*; crab *Portunus sanguinoletus*) and two mollusc species (gastropod *Babylonia areolata*; bivalve *Paphia amabilis*) were assigned randomly to air and ice treatments (*n* = 4 per species per treatment). The air treatment quantified the extent of natural decomposition, while the ice treatment evaluated the effectiveness of using an ice covering to reduce any decomposition effects. Repeated tissue sampling per individual was undertaken at 0, 30, 60, 90 and 120 h. Repeated measures within individuals were used because the results of a pilot experiment suggested that large between-individual variations in derived response variables might mask decomposition effects. Fish and mantis shrimp remained whole, with dorsal muscle and abdominal muscle, respectively, sampled at each time point. Due to difficulties of separating tissues because of decomposition / drying over time, crabs and molluscs were completely dissected fresh (Time = 0 h), with claw and leg muscle (crab), foot muscle (gastropod), or foot and adductor muscle (bivalve) pooled per individual on a petri dish from which subsequent samples were taken over time. Both air and ice treatments provided aerobic conditions. ‘Air’ treatment samples were kept in open trays within the laboratory (mean ± SD across experiment 1 and 2: temperature = 20.91°C ± 0.81°C, *n* = 10; humidity = 69.82% ± 3.22%, *n* = 10) while ‘ice’ treatment samples were kept in open freezer bags placed within ice (0.08°C ± 0.86°C, *n* = 10; humidity = 50.65% ± 2.10%, *n* = 10). Collected samples were immediately freeze dried for >48 h, homogenized and stored under dry conditions. For each of the freeze dried samples, 1.00mg ± 0.10mg dried tissue was carefully weighed and enclosed in a tin capsule and analysed for *δ*^15^N, *δ*^13^C, %N, and %C using stable isotope ratio mass spectrometry (EuroVector, model EA3028) at the Stable Isotope Laboratory of the Science Faculty, University of Hong Kong. For each sample, C:N ratio was subsequently calculated by dividing %C by %N. Further details can be found in S1 File.

#### Lipid correction

Following Post et al. [26], we applied an *a posteriori* mathematical correction to all estimates of *δ*^13^C for fish and crustacean samples to provide lipid free estimates of *δ*^13^C. Corrections were based on derived equations of relationships between C:N ratio and the difference in *δ*^13^C with and without lipid (Table A in S1 File). Thus, for fish and crustaceans, we assessed tissue decomposition over time in air and ice upon *δ*^13^C from tissues with lipid (termed *δ*^13^C +L) and for values of *δ*^13^C that were mathematically corrected to account for lipids (termed *δ*^13^C -L). It was not possible to derive such relationships for mollusc tissues, so for molluscs we only investigated effects of decomposition on uncorrected *δ*^13^C (*δ*^13^C +L) and interpreted the results with caution.

#### Data analysis

All statistical analyses were performed in the open source software R 3.4.0 [28] using lmer in lme4 [29]. We used a separate analysis per species per response variable. Per species, we used a linear mixed-model (LMM) to test for an interaction between the fixed effects of *time* (continuous variable) and *treatment* (2 level factor: air and ice), separately, on *δ*^*15*^*N*, *δ*^*13*^*C +L*, *δ*^*13*^*C -L*, *%N*, *%C* and *C*:*N ratio*. We included the random effect *individual* in each model to account for non-independence of repeated measures over time per individual. Significance of an interaction effect was determined using analysis of deviance between models with and without the interaction included. Interactions were dropped when they were not significant, and main effects were similarly tested by analysis of deviance between models with and without each term. For LMM, determination of model fit to the data was calculated with pseudo-*R* squared values [30] using the R package MuMIn [31], with estimates for the variance explained by model fixed effects reported with the prefix *R*^2^_GLMM(m)_ and the combined fixed and random effects as *R*^2^_GLMM(c)_. For all analyses, normality of residuals was checked visually using Q-Q plots, and homogeneity of variances checked visually using plots of residuals against each variable.

### Experiment 2

Questions 2 and 3 are both tested using a common dataset derived from the experimental design below.

#### Sample preparation

In contrast to experiment 1, experiment 2 used only fish. This was because a pilot experiment suggested individual fish (c.f. mollusc and crustaceans) provided ample tissue for multiple lipid quantifications, while different fish species provided a broad range of lipid contents against which to test the effects of tissue decomposition. Using six species of fishes (seabream *Acanthopagrus latus*; grouper *Epinephelus lanceolatus* (hybrid); pomfret *Pampus argenteus*; scat *Scatophagus argus*; rabbitfish *Siganus canaliculatus* and mullet *Valamugil cunnesius*), one individual per species was assigned to each the air and ice treatment. For all individuals, repeated tissue samples (dorsal muscle) were collected at 0, 60 and 120 h. Thus, each level of treatment and time (Air 0h, A 60h, A 120h, Ice 0h, I 60h, I 120h) had a sample size of six (one sample per the single individual of each of the six fish species). Fish were kept in air or ice as described for Experiment 1 prior to sampling, where upon tissues were immediately freeze dried, homogenised and stored under dry conditions. For each sample we quantified lipid content, C:N ratio and *Δδ*^13^C. *Δδ*^13^C was calculated as the difference in *δ*^13^C between one sub-sample that underwent stable isotope analysis (SIA) and a second sub-sample that underwent lipid extraction and then SIA. Therefore, subsequent relationships between (i) lipid content and *Δδ*^13^C, (ii) C:N ratio and lipid content, and (iii) C:N ratio and *Δδ*^13^C were examined at each level of treatment and time. Note, it is appropriate to test these relationships across individuals of different species (c.f. across individuals within species) to maximise range of lipid content [26].

#### Lipid extraction & quantification

Samples for each individual per time and treatment underwent lipid extraction and quantification as described by Post et al. [26]. Briefly, 0.5g ± 0.0001g of homogenised dried tissue was placed in a 30ml boiling-tube to which 8ml of chloroform and 8ml of methanol were added, forming a 50:50 methanol-chloroform solution. Boiling tubes were heated in a water bath at 61°C for 40 min, allowed to cool to air temperature, and refilled to 25ml with the addition of chloroform. The mixture was poured through a No. 1 Whatman filter paper into a 250ml separatory funnel. 10ml of 0.9% saline solution was added to the separatory funnel, and the whole mixture shaken vigorously and allowed to settle and separate for 45min. The bottom methanol-chloroform fraction containing the lipid was drained into a pre-weighed aluminium dish and warmed on a hot-plate at 70°C until no liquid remained. The dish was cooled to air temperature, re-weighed to the nearest 0.0001g, and the mass of lipid calculated as the difference between post and pre tray weight, and expressed as a percentage of the original 0.5g sample mass, termed ‘lipid content’. The residual sediment on the filter paper was air dried and collected for use as the lipid extracted sample for SIA.

#### Stable isotope analysis

All samples were analysed for *δ*^15^N, *δ*^13^C, %N, %C and C:N ratio using stable isotope ratio mass spectrometry as described for Experiment 1.

#### Data Analysis ― *Q2*

**Does tissue decomposition affect tissue lipid content or relationships between (i) lipid content and *Δδ***^**13**^**C, (ii) C:N ratio and lipid content, and (iii) C:N ratio and *Δδ***^**13**^**C?** Firstly, we used a LMM to test if *time*, *treatment* (2 level factor: air and ice) and their interaction affected *lipid content* of fish tissues, and included the random effect *individual* to account for non-independence of repeated measures over time per individual fish. Next, for three known relationships between (i) lipid content and *Δδ*^13^C, (ii) C:N ratio and lipid content, and (iii) C:N ratio and *Δδ*^13^C, separately, we investigated how each relationship was affected by tissue decomposition in air or ice. To simplify the analyses, we combined *treatment* and *time* to produce a single variable termed *combined treatment* (6 level factor: Air 0h, A 60h, A 120h, Ice 0h, I 60h, I 120h). Using separate LMMs, we tested if (i) *Δδ*^*13*^*C* was affected by *lipid content*, *combined treatment* and their interaction, (ii) *lipid content* was affected by *C*:*N ratio*, *combined treatment* and their interaction, and (iii) *Δδ*^*13*^*C* was affected by *C*:*N ratio*, *combined treatment* and their interaction. The random effect *individual* was included in each model to account for non-independence of repeat measures over time per individual fish. Model selection and estimates of model fit were conducted for all analyses as previously described for Experiment 1.

#### *Q3*. Are alternative methodologies for estimating *δ*^13^C differentially affected by tissue decomposition?

Finally, we tested how tissue decomposition may affect *δ*^13^C values for samples prepared using different methodological procedures. At each sampling event (0, 60 and 120 h) per individual fish, we derived three measures of *δ*^13^C: from a sample that underwent SIA (termed uncorrected), a sample that underwent SIA and then *a posteriori* mathematical correction (termed mathematically corrected), and a sample that underwent lipid extraction and then SIA (termed lipid extracted). Mathematical correction was achieved using samples’ C:N ratios and equation 3 in Table A in S1 File. Given potentially larger between-individual than within-individual variation in derived *δ*^13^C values, to aid direct comparison of *δ*^13^C values derived across individuals over time and from multiple methodological procedures, we firstly standardised all values of *δ*^13^C. Within each individual fish, standardisation was achieved by subtracting all sampled values of *δ*^13^C from a ‘baseline *δ*^13^C’ which was taken as each fish’s value of lipid extracted *δ*^13^C at 0 h. Lipid extracted values were deemed to be the most accurate estimates of a lipid-free ‘true’ *δ*^13^C value, while using 0 h tissues ensured no decomposition had occurred. The difference between sample *δ*^13^C and baseline *δ*^13^C is hereafter termed *standardised δ*^*13*^*C*. Lipid extracted values of *δ*^13^C at 0 h were, therefore, not included in subsequent analysis, given all other values of *δ*^13^C were standardised against them. As previously, to simplify the analysis, we combined *treatment* (2 level factor: air or ice) and *time* to produce a single variable termed *combined treatment* (6 level factor for uncorrected and mathematically corrected samples: Air 0h, A 60h, A 120h, Ice 0h, I 60h, I 120h; but a 4 level factor for lipid extracted samples: A 60h, A 120h, I 60h, I 120h). Using a separate analysis for each methodological procedure, a LMM tested if *standardised δ*^*13*^*C* was affected by the main effect *combined treatment*, while the random effect *individual* was included in each model to account for non-independence of repeat measures over time per individual fish. Model selection and estimates of model fit were conducted as previously described for Experiment 1. Following a significant result, post-hoc analysis was used to determine differences between levels of factors using a *P*-value adjusted Tukey method (to account for multiple testing) in the R package lsmeans [32]. For all analyses, homogeneity of variances and normality of model residuals were checked as described in Experiment 1. All statistical analyses in questions 2 and 3 were performed in the open source software R 3.4.0 [28] using lme4 [29].

## Results

### Experiment 1

**Fig 1:**
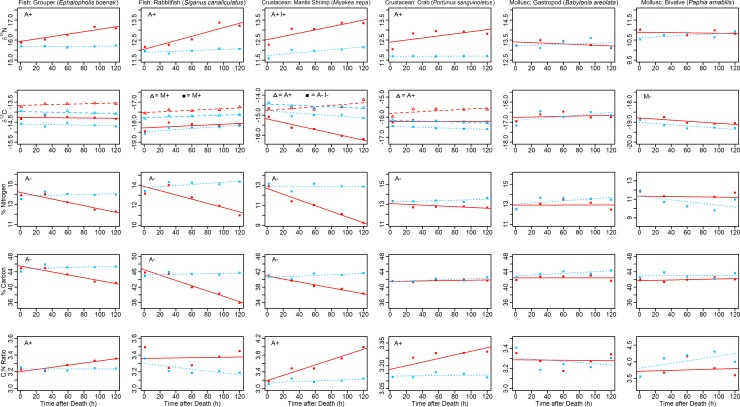
Change in *δ*^*15*^*N*, *δ*^*13*^*C*, *%N*, *%C* and *C*:*N ratio* over 120 h for fish, crustacean and mollusc tissues kept in air (solid red lines) or ice (dotted blue lines). Slopes are modelled relationships, and data represent mean values (*n* = 4). Error bars are not included as they are not appropriate here; between-individual variation was excluded from models using repeated measures within individuals and accounted for in analyses through use of a random effect ‘*individual*’. For *δ*^*13*^*C*, all species use *δ*^*13*^*C* +L (square symbols with either solid red or dotted blue lines), while we also modelled *δ*^*13*^*C* -L for fish and crustaceans (open triangle symbols with either red or blue dashed lines). *δ*^*13*^*C* -L values were obtained by applying a mathematical correction (Table A in S1 File) to *δ*^*13*^*C* +L data. Significant effects of tissue decomposition over time upon tissue measures are denoted for air (A) and ice (I) treatments where interactions indicated differences between treatments, or (M) when determined by a significant main effect indicating tissue decomposition that did not differ between treatments. + /—indicates the direction of effect. For fish and crustacean *δ*^*13*^*C*, symbol type with letter indicates significant relationships for either *δ*^*13*^*C* -L (open triangles) or *δ*^*13*^*C* +L (squares).

***Q1*. How does tissue decomposition affect *δ***^**15**^**N, *δ***^**13**^**C, %N, %C and C:N ratio?** Across all species and response variables, 60% of analyses (18 of 30; Fig 1) showed a significant change over time. Tissue decomposition frequently altered tissue values in air (18 of 30) but infrequently in ice (3 of 30). Decomposition caused *δ*^15^N and *δ*^13^C to become enriched, caused decreases in both %N and %C, and subsequently increased C:N ratio (inferring decreases in %N were proportionally larger than decreases in %C). Broadly consistent patterns of change were observed for fish and crustacean tissues, which contrasted with the detection of only one significant change in mollusc tissues (1 in 10 analyses). Given the large non-directional variances seen within individual mollusc tissues regardless of the treatment (Fig A in S1 File), this suggested that within individual variability may have masked smaller effects of decomposition. Excluding molluscs, we observed a significant effect of tissue decomposition on the five measured parameters in 85% of cases (17 of 20; Fig 1). Notable results are presented per tissue measure below, with further details in S1 File.

***δ***^**15**^**N.** For fish and crustacean analyses, significant interactions indicated that *time* affected *δ*^15^N but that this was dependent upon *treatment* (Grouper: *χ*^2^_(1)_ = 17.91, *P* < 0.001, *R*^2^_GLMM(m)_ = 0.37; Rabbitfish: *χ*^2^_(1)_ = 20.10, *P* < 0.001, *R*^2^_GLMM(m)_ = 0.63; Mantis Shrimp: *χ*^2^_(1)_ = 6.88, *P* < 0.01, R^2^_GLMM(m)_ = 0.68; Crab: *χ*^2^_(1)_ = 7.74, *P* < 0.01, *R*^2^_GLMM(m)_ = 0.66; Fig 1; Table B in S1 File). For these species, tissues kept in air enriched in *δ*^15^N between 0.6‰ to 1.3‰ over 120 h (Fig 1; Table B in S1 File). Conversely, *δ*^15^N of tissues kept in ice did not change over time, except for Mantis Shrimp, which were modestly enriched in *δ*^15^N by 0.4‰ over 120 h (Fig 1; Table B in S1 File).

***δ***^**13**^**C.** Using *δ*^13^C -L data, we observed that decomposition over time caused *δ*^13^C to become significantly enriched in air (Rabbitfish, Mantis Shrimp and Crab) and in ice (Rabbitfish only), though this change was small: 0.2‰ to 0.4‰ over 120 h (Fig 1; Table C in S1 File). For Rabbitfish, this was determined as a main effect of *time* (*χ*^2^_(1)_ = 9.07, *P* < 0.01, *R*^2^_GLMM(m)_ = 0.02; Table C in S1 File) indicating *δ*^13^C was enriched in both air and ice, while for Mantis Shrimp and Crab, significant interactions provided evidence of change in *δ*^13^C over *time* that was dependent upon *treatment* (Mantis Shrimp: *χ*^2^_(1)_ = 8.80, *P* < 0.01, *R*^2^_GLMM(m)_ = 0.13; Crab: *χ*^2^_(1)_ = 9.88, *P* < 0.01, *R*^2^_GLMM(m)_ = 0.59; Table C in S1 File). Conversely, for molluscs we only had *δ*^13^C +L data, and we observed decomposition over time caused *δ*^13^C to become significantly depleted in both air and ice (Bivalve only) by 0.3‰ over 120 h (main effect of *time*: *χ*^2^_(1)_ = 16.40, *P* < 0.001, *R*^2^_GLMM(m)_ = 0.13; Fig 1; Table D in S1 File). However, we suspected this result was likely due to lipid affecting Bivalve *δ*^13^C values. This was supported by the crustacean results, which showed tissue decomposition in air depleted *δ*^13^C (Mantis Shrimp) or had no effect (Crab) when using *δ*^13^C +L data (Fig 1; Table D in S1 File) but enriched *δ*^13^C when using *δ*^13^C -L data (Fig 1; Table C in S1 File). Thus, these conflicting results clearly demonstrate the need to account for lipids to accurately estimate *δ*^13^C and thus, that only our *δ*^13^C results based on *δ*^13^C -L data are reliable.

**%N.** For fish and crustacean analyses, significant interactions indicated that *time* affected %N but that this was dependent upon *treatment* (Grouper: *χ*^2^_(1)_ = 16.17, *P* < 0.001, *R*^2^_GLMM(m)_ = 0.59; Rabbitfish: *χ*^2^_(1)_ = 23.38, *P* < 0.001, *R*^2^_GLMM(m)_ = 0.71; Mantis Shrimp: *χ*^2^_(1)_ = 29.30, *P* < 0.01, *R*^2^_GLMM(m)_ = 0.78; Crab: *χ*^2^_(1)_ = 7.07, *P* < 0.01, *R*^2^_GLMM(m)_ = 0.52; Fig 1; Table E is S1 File). For these species, tissues kept in air showed decreases in %N ranging from 0.47 to 3.43 percentage points over 120 h (c.f. starting values at 0 h of ~13%), while %N of tissues kept in ice did not change over time (Fig 1; Table E in S1 File).

**%C.** For fish and Mantis Shrimp analyses, significant interactions indicated that *time* affected %C but that this was dependent upon *treatment* (Grouper: *χ*^2^_(1)_ = 11.20, *P* < 0.001, *R*^2^_GLMM(m)_ = 0.48; Rabbitfish: *χ*^2^_(1)_ = 42.33, *P* < 0.001, *R*^2^_GLMM(m)_ = 0.85; Mantis Shrimp: *χ*^2^_(1)_ = 25.92, *P* < 0.001, *R*^2^_GLMM(m)_ = 0.66; Fig 1; Table F in S1 File). For these species, tissues kept in air showed decreases in %C ranging from 4.53 to 8.29 percentage points over 120 h (c.f. starting values at 0 h of ~43%), while %C of tissues kept in ice did not change over time (Fig 1; Table F in S1 File).

**C:N ratio.** For Grouper and crustacean analyses, significant interactions indicated that *time* affected C:N ratios but that this was dependent upon *treatment* (Grouper: *χ*^2^_(1)_ = 5.64, *P* < 0.05, *R*^2^_GLMM(m)_ = 0.30; Mantis Shrimp: *χ*^2^_(1)_ = 17.95, *P* < 0.001, *R*^2^_GLMM(m)_ = 0.71; Crab: *χ*^2^_(1)_ = 11.16, *P* < 0.001, *R*^2^_GLMM(m)_ = 0.68; Fig 1; Table G in S1 File). For these species, in each instance, tissues kept in air showed increases in C:N ratios ranging from 0.14 to 0.75 over 120 h, while tissues kept in ice did not change (Fig 1; Table G in S1 File). Increases in C:N ratio suggested greater proportional deceases in %N than %C when compared to their respective starting values at 0 h, despite absolute decreases in percentage points (reported above) being greater in %C than %N. The magnitude of changes in C:N ratios should be compared with a mean C:N ratio across all species at 0 h of 3.31 ± 0.22 SD.

### Experiment 2 ― *Q2*. Does tissue decomposition affect tissue lipid content or relationships between (i) lipid content and *Δδ*^13^C, (ii) C:N ratio and lipid content, and (iii) C:N ratio and *Δδ*^13^C?

#### Lipid content

Across all samples, lipid content ranged between 3.27% and 29.20% (Mean ±SD = 14.55% ± 7.39). Analysis revealed *lipid content* of fish tissues was not affected by the interaction (χ^2^_(1)_ = 3.33, *P* = 0.07) or main effects of *time* (χ^2^_(1)_ = 1.34, *P* = 0.25) and *treatment* (χ^2^_(1)_ = 1.93, *P* = 0.16) (Table H in S1 File). Thus, observed changes in lipid content with time (between 0 and 120 h) were inconsistent between individuals and across species. Variation in lipid content across repeated measures within individuals ranged between 2.97% to 12.14%.

#### Relationships between C:N ratio, lipid content and *Δδ*^13^C

**Fig 2:**
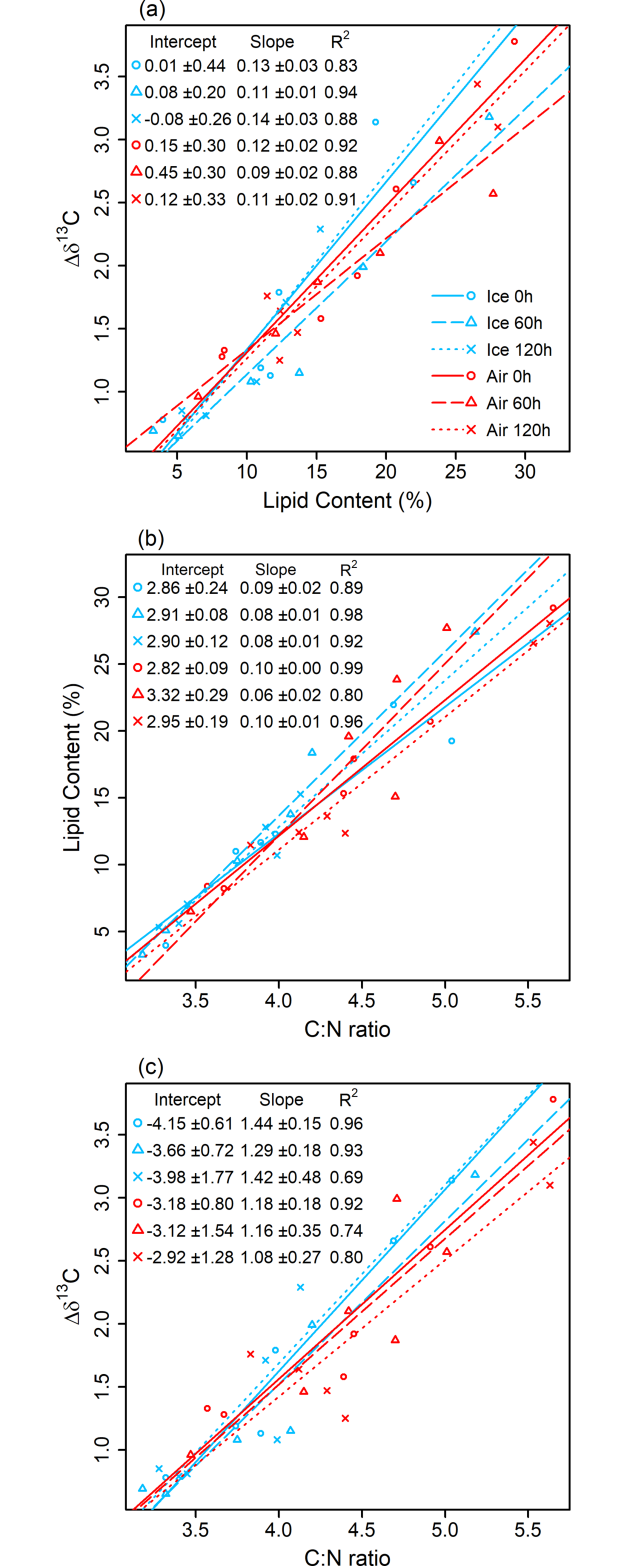
**Significant relationships between (a) *lipid content* and *Δδ***^***13***^***C* (b) *C*:*N ratio* and *lipid content* and, (c) *C*:*N ratio* and *Δδ***^***13***^***C* of fish muscle tissues were not affected by exposure to air or ice over 120 h.** Per (a), (b) and (c), modelled slopes and raw data for each exposure time (0, 60 and 120 h) in air (red lines) and ice (blue lines) are shown, although they do not significantly differ from one another. Model estimates of slopes, intercepts and model variance explained (*R*^2^) are also given.

LMMs tested if relationships between (i) lipid content and *Δδ*^13^C, (ii) C:N ratio and lipid content, and (iii) C:N ratio and *Δδ*^13^C were significantly different across six levels of the composite variable ‘*combined treatment*’ (6 level factor: Air 0h, A 60h, A 120h, Ice 0h, I 60h, I 120h). Per each relationship in (i), (ii) and (iii), *combined treatment* was not significant either as an interaction (*χ*^2^_(5)_ = 7.72, *P* = 0.17; *χ*^2^_(5)_ = 10.19, *P* = 0.07; *χ*^2^_(5)_ = 8.40, *P* = 0.14, respectively) or main effect (*χ*^2^_(5)_ = 10.30, *P* = 0.07; *χ*^2^_(5)_ = 7.57, *P* = 0.18; *χ*^2^_(5)_ = 7.44, *P* = 0.19, respectively). Thus, our analyses did not detect any evidence for tissue decomposition affecting significant relationships between lipid content and *Δδ*^13^C (*χ*^2^_(1)_ = 55.84, *P* < 0.001, *R*^2^_GLMM(m)_ = 0.88, intercept ± SE = 0.26 ± 0.13, slope ± SE = 0.10 ± 0.01), C:N ratio and lipid content (*χ*^2^_(1)_ = 63.51, *P* < 0.001, *R*^2^_GLMM(m)_ = 0.92, intercept = -28.63 ± 2.37, slope = 10.27 ± 0.56), C:N ratio and *Δδ*^13^C (*χ*^2^_(1)_ = 61.40, *P* < 0.001, *R*^2^_GLMM(m)_ = 0.84, intercept = -2.79 ± 0.36, slope = 1.09 ± 0.08). Though no significant differences were detected between slopes and intercepts of levels of *combined treatment*, we report the individual *combined treatment* levels for relationships (i), (ii) and (iii) in Fig 2.

**Fig 3:**
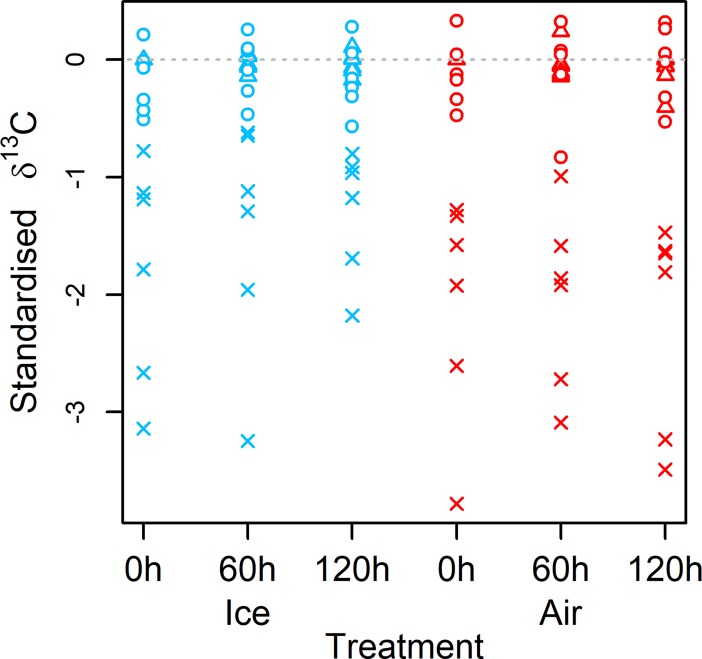
Standardised *δ*^13^C of fish muscle tissue was not affected by tissue decomposition over 120 h in either air or ice, but was dependent upon methodological procedure. Standardised *δ*^13^C of a sample was the difference in the sample’s *δ*^13^C from a lipid extracted value of *δ*^13^C at 0 h, termed baseline *δ*^13^C. Baseline *δ*^13^C is indicated as a dashed grey line at y = 0. Standardised *δ*^13^C of lipid extracted (triangles) and *a posteriori* mathematically corrected (circles) samples did not differ from one another and were typically small (range -0.8‰ to 0.3‰). By contrast, standardised *δ*^13^C of uncorrected samples (crosses) were significantly depleted (range -0.6‰ to -3.8‰) compared to lipid extracted and mathematically corrected samples.

***Q3*. Are alternative methodologies for estimating *δ***^**13**^**C differentially affected by tissue decomposition?** Separate analyses per methodological procedure using LMMs showed that tissue decomposition over time in either air or ice did not affect estimates of *standardised δ*^*13*^*C* (the difference between sample *δ*^13^C and baseline *δ*^13^C) derived from samples that were uncorrected (*χ*^2^_(5)_ = 9.41, *P* = 0.09), mathematically corrected (*χ*^2^_(5)_ = 3.90, *P* = 0.56) or lipid extracted (*χ*^2^_(3)_ = 2.07, *P* = 0.56). Subsequently, all data was pooled, and using a LMM we observed that *methodological procedure* (3 level factor: uncorrected, mathematically corrected, lipid extracted) significantly affected *standardised δ*^*13*^*C* (*χ*^2^_(2)_ = 134.59, *P* < 0.001, *R*^2^_GLMM(m)_ = 0.69). Post-hoc analysis revealed that *standardised δ*^*13*^*C* of uncorrected samples was significantly lower than mathematically corrected (difference ± SE = 1.7‰ ± 0.1, t.ratio = 16.07, df = 84, *P* < 0.001) and lipid extracted (difference ± SE = 1.8‰ ± 0.1, t.ratio = 14.92, df = 84, *P* < 0.001), while mathematically corrected and lipid extracted samples did not differ (difference ± SE = 0.1‰ ± 0.1, t.ratio = 0.55, df = 84, *P* = 0.85) (Fig 3). Thus, lipid extraction and mathematical correction both produced estimates of *δ*^13^C that approximated a *δ*^13^C baseline, while the use of uncorrected samples (where lipid content was not removed or accounted for) resulted in *δ*^13^C estimates that were between 0.6‰ and 3.8‰ depleted compared to the baseline value of *δ*^13^C.

## Discussion

### Overview

In our first experiment (question 1), over 120 h we observed changes in tissues of selected marine animals due to decomposition in air, but only rarely in ice. The latter was effective at minimizing tissue decomposition over this period. Decomposition of tissues in air caused *δ*^15^N and *δ*^13^C to become enriched, and caused proportionally greater decreases in %N relative to decreases in %C, subsequently increasing C:N ratio. Given that step-wise enrichment in *δ*^15^N from source to consumer is used to calculate consumer trophic levels and food chain length, so the largest changes in *δ*^15^N were equivalent to half a trophic level, while C:N ratios (when expressed as a proportion of C:N ratio at 0 h) showed notable increases up to 23%. Thus, alteration of *δ*^15^N and C:N ratio by decomposition was non-trivial and has the potential to affect subsequently derived results and conclusions, inferring decomposed tissue values should be interpreted with caution. In contrast, changes in *δ*^13^C were minor, suggesting it is likely to be acceptable to use *δ*^13^C signatures derived from decomposed (~120 h) samples if necessary.

Our second experiment (question 2) demonstrated that decomposition did not affect the use of relationships between C:N ratio, lipid content and *Δδ*^13^C to derive accurate estimates of *δ*^13^C within fish tissues, inferring that any effects of tissue decomposition remained proportional across these measures. Therefore, if a researcher was faced with samples that had been exposed to air for up to 120 h, our findings suggest that a sub-set of those samples could be subjected to lipid extraction and quantification and used to derive an accurate mathematical correction to account for the effects of lipid on *δ*^13^C. Additionally, our second experiment (question 3) clearly demonstrated that lipids caused *δ*^13^C to be significantly depleted (0.6‰ to 3.8‰), constituting error for isotope-based studies. This result puts into context the minor effects of tissue decomposition compared with the important need to account for lipids in samples to attain accurate *δ*^13^C values. Notably, both lipid extraction and mathematical correction performed equally well to correct for lipids when estimating *δ*^13^C.

Currently, freezing or drying are recommended as effective means to preserve stable isotope signatures of samples in the long-term [5,7,13], despite changes to signatures in a few instances following freezing [7,15,16]. From a practical perspective, our results demonstrate the effectiveness of ice as an important ‘bridge’ for preserving tissue measures such as stable isotope signatures and C:N ratios in the short-term, which may be necessary when pre-sorting and identifying large numbers of samples prior to long-term freezing / drying.

### Experiment 1

***Q1*. How does tissue decomposition affect *δ***^**15**^**N, *δ***^**13**^**C, %N, %C and C:N ratio?** Results of studies examining various derived measures of nitrogen and carbon in tissues subjected to decomposition have shown mixed results, suggesting highly species and context specific changes [17,18,21,33–35]. A general understanding of decomposition processes affecting tissues measures is, therefore, still emerging.

Our results suggest that decomposition of tissues proceeds through physical loss of nitrogen and carbon, observed as decreases in both %N and %C (Fig 1), in agreement with previous studies examining early- and late-stages of animal and early-stages of plant decomposition which showed net loss of nitrogen and/or carbon mass from marine vertebrates [21] and seagrasses and mangroves [33,35]. Our first experiment demonstrated such losses can be notable; when expressed as percentage change from the starting value at 0 h, losses after 120 h in air ranged between 3.53% and 26.58% for %N (note the absolute decreases in %N were 0.47 to 3.43 percentage points) and between 11.22% and 18.10% for %C (note the absolute decreases in %C were 4.53 to 8.29 percentage points). The shift to higher C:N ratios over time in air observed in our study is likely attributable to a greater proportional loss of %N than %C (Fig 1), despite percentage point losses of %C tending to be greater than %N. When expressed as a proportion of C:N ratio at 0 h, such changes were again considerable, being between 4.56% and 23.70% (note the absolute increases in C:N ratio were 0.14 to 0.75). Increases in C:N ratio over time were linear, inferring decomposition began immediately and greater changes would be expected after the 120 h period our experiment covered.

To date, studies using experimental decomposition of animal tissues to examine changes in nitrogen and carbon content have been scarce. Significant decreases in %N and %C (percentage points; N: 0.47 to 3.43; C: 4.53 to 8.29) of fish and crustacean muscle tissues over 5 days as observed in our study, are concurrent with smaller significant decreases (percentage points; N: 0.4 to 1.9; C: 3.3 to 7.3) within fish and shark muscle tissue over 256 days [21]. Conversely, observed significant increases in C:N ratio in our study (0.14 to 0.75) contrast with observations of no change for fish, shark and seal muscle tissue over 256 days [21]. Determination of mechanisms underlying tissue decomposition is beyond the scope of this study, but speculatively, the larger changes in %N and %C content, and change in C:N ratios, observed in a shorter-time period in our study, maybe reflective of different tissue sampling techniques between studies. For many species, we decomposed entire animals from which we re-sampled at each time point, whereas Yurkowski et al. [21] report dissecting all samples prior to the start of their experiment. In the latter study, smaller samples that were isolated from surrounding body tissue may have dried relatively more quickly so limiting change in tissue qualities. Indeed, we noted that odour associated with decomposition was reduced as specimens became increasing dry at later stages of our experiment, suggesting decay is associated with aridity, concurrent with findings in Yurkowski et al. [21].

Relative losses of carbon to nitrogen, or crudely lipid to protein, are dependent upon environmental context and specific to micro-organism activity based partly on available amino acids in substrate tissues [18,36]. Greater utilisation of nitrogen from the source tissue by micro-organisms is predicted when tissue C:N ratios are lower [37], with animal tissues having lower C:N ratios relative to plant tissues [38]. Enriched *δ*^15^N with decomposition of animal tissues observed in our study, and concurrent with previous work [13,21] may therefore be due to preferential loss from tissues of lighter ^14^N representing more easily degradable compounds altered and/or assimilated by microbial activity. This result contrasts with previous studies on degrading plant materials that observed depleted *δ*^15^N [18,33] or no change [33,35]. Higher C:N ratios in plant than animal tissues [38] may provide a more limited nitrogen pool where micro-organism driven processes may strongly influence nitrogen content (even increasing it through immobilization of environmental nitrogen) [39], thus potentially influencing *δ*^15^N, and whereby carbon losses may be greater relative to nitrogen as observed by decreases in C:N ratio with decomposition [18,37,39].

Importantly, the magnitude to which *δ*^15^N enriched in our study was up to 1.3‰ in air over 120 h (Fig 1, Table B in S1 File), though again the relationship was linear such that change could be greater over longer periods (Yurkowski et al. [21] observed *δ*^15^N of marine vertebrates enriched up to 2.2‰ over 256 days). Given that trophic discrimination of *δ*^15^N (the change in *δ*^15^N from resource to consumer) enriches per trophic transfer in the region of 2.5‰ to 3.4‰ [12,40,41], the largest changes we observed (~1.3‰) due to tissue decomposition may be equivalent to half a trophic level. Short-term decomposition in animal tissues has been scarcely documented; concurrent with our findings, decomposition of skin from *Orca* exposed in air at 20°C for 14 days also showed enriched *δ*^15^N of 6.4‰ [20], while trout muscle enriched in air by 0.4‰ over 8 days [21].

Our results also showed tissue decomposition enriched *δ*^13^C, after accounting for lipids (*δ*^13^C -L). Enriched *δ*^13^C with decomposition may be due to preferential loss from tissues of lighter ^12^C representing more easily degradable compounds, via microbial use or direct oxidation [17,35]. The magnitude of change in air was limited to 0.4‰ over 120 h (Fig 1, Table C in S1 File). Tarroux et al. [11] showed error in *δ*^13^C of ~2‰ caused spurious dietary estimates using mixing models, suggesting that decomposition error as we observed would likely introduce only minor bias into dietary analyses. Concurrent with our findings, decomposition of skin from *Orca* exposed in air at 20°C for 14 days enriched in *δ*^13^C by 1‰ [20], although other marine vertebrates have shown variable results, with cetacean and turtle tissues showing no changes over 62 days [19] and fish, shark and seal being enriched or depleted ≤ 0.8‰ after 256 days [21]. Elsewhere, studies have shown highly species-specific changes in *δ*^13^C for marine algae and plants, with no change [33,35] or either enriched or depleted *δ*^13^C of around 1‰ [18,34]. Therefore, further research is required to determine the mechanisms underlying such species- or tissue-specific differences in decomposition changes in *δ*^13^C.

### Experiment 2

***Q2*. Does tissue decomposition affect tissue lipid content or relationships between (i) lipid content and *Δδ***^**13**^**C, (ii) C:N ratio and lipid content, and (iii) C:N ratio and *Δδ***^**13**^**C?** As shown in this study, fish tissue lipid content was not affected by decomposition in either air or ice (Table H in S1 File). Non-directional changes in lipid content were observed within individuals (mean within-individual variance: 6.15% ± 2.71% SD), suggesting that within tissue variation was greater than any effects of tissue decomposition. Such within-individual changes in lipid content, either due to natural tissue lipid variation or undetected decomposition, remained proportional to both C:N ratio and *Δδ*^13^C; exposure to air or ice over 120 h did not affect linear relationships between (i) lipid content and *Δδ*^13^C, (ii) C:N ratio and lipid content, and (iii) C:N ratio and *Δδ*^13^C (Fig 2). Furthermore, for all three linear relationships, tissue decomposition did not affect the quantity of data variance explained by the relationships (range of *R*^2^ across time in air and ice remained high for all relationships: 0.69 to 0.99, Fig 2). Therefore, linear relationships between C:N ratio, lipid content and *Δδ*^13^C derived from tissues exposed in air for 120 h would provide accurate mathematically corrected estimates of *δ*^13^C. However, we advise the precautionary use of ice to ensure preservation of samples whenever possible. Notably, the range of C:N ratios in the present study (~ 3 to 6) ranged comparably to those of a key study reporting this mathematical correction method (~ 3 to 7; [26]), representing lipid content ranges from ~ 3% to 30%. Thus, our study provided a relevant test of the robustness of this widely applied *a posteriori* mathematical correction to the potential effects of tissue decomposition.

***Q3*. Are alternative methodologies for estimating *δ***^**13**^**C (SIA; lipid extraction then SIA; SIA then mathematical correction) differentially affected by tissue decomposition?** Our study additionally compared potential decomposition effects on three different methods of estimating *δ*^13^C (SIA; *a posteriori* mathematical correction of SIA values; samples subjected to lipid extraction prior to SIA). Tissue decomposition in air or ice over 120 h did not affect estimates derived from each method (Fig 3). Our first experiment demonstrated that *δ*^13^C derived from SIA, or from *a posteriori* mathematical correction of SIA values, may change over time in response to tissue decomposition (Fig 1, Tables C and D in S1 File). However, such change was typically small (≤ 0.4‰), while not all species showed change. Therefore, there was no expectation that *δ*^13^C derived from SIA or *a posteriori* mathematical correction in experiment 2 would necessarily show effects of tissue decomposition, and so the results of our experiments 1 and 2 are concurrent.

A further finding of this analysis was that *δ*^13^C values derived from different methodological procedures differed significantly. Compared with a lipid-free baseline *δ*^13^C value at 0 h, uncorrected SIA *δ*^13^C values were significantly depleted in *δ*^13^C (mean value ± SE = -1.8‰ ± 0.1) which contrasted to either mathematically corrected (-0.1‰ ± 0.0) or lipid extracted samples (0.0‰ ± 0.0) which were not (Fig 3). This is likely attributable to lipid contained within the uncorrected samples. Lipid is naturally depleted in *δ*^13^C [27] and its content varies between tissues and individuals [10]. Most ecological studies using isotopic data aim to derive feeding relationships or food web structure using assumed step-wise enrichment in *δ*^13^C from source to consumer, so the inclusion of lipid in estimates of *δ*^13^C may constitute error. It has been shown that error of ~2‰ in *δ*^13^C of sources or consumers may cause spurious conclusions when estimating diets using mixing models [11]. Therefore, our use of a lipid-free baseline at 0 h, and the near exact matching of that baseline by other lipid extracted samples (at 60 or 120 h) and mathematically corrected samples (which had not had lipids removed, at 0, 60 and 120 h), highlights the effectiveness of these techniques to derive lipid-free estimates of *δ*^13^C. It also demonstrates the potentially large errors in *δ*^13^C when not correcting for lipid (Fig 3). The need to account for lipids in samples when estimating *δ*^13^C has been previously highlighted [10,11,26], yet many studies still do not account for lipids or report doing so. Explicit demonstration of the large discrepancies in *δ*^13^C estimates for tissues when failing to account for lipids is thus a further important aspect of this present study, as we demonstrated that the decomposition error on *δ*^13^C (as shown in experiment 1) was much smaller when compared with error caused by lipids.

### Conclusions

Firstly, our results show that tissue decomposition over the short-term in air can cause notable changes to measures of *δ*^15^N, %N, %C and C:N ratio (but less so *δ*^13^C), but that storing specimens in ice is effective in mitigating such changes to levels that are likely to have little impact upon results and conclusions of these parameters. Secondly, our results suggest that short-term tissue decomposition in air or ice has a negligible influence on the relationships between lipid content, C:N ratio, and Δδ^13^C in marine fishes. Nonetheless, we advise that ice should be used for short-term sample preservation to ensure that the relationship between C:N ratio and Δδ^13^C can be accurately applied as a mathematical correction to estimates of δ^13^C.

## Supporting information

S1 File Appendix contains additional information on methods and results associated with this study (Tables A-H and Fig A). (DOCX)

S2 File Excel dataset contains all primary data used in this study. (XLSX)
