# Supplementary material for: Short-term tissue decomposition alters stable isotope values and C:N ratio, but does not change relationships between lipid content, C:N ratio, and Δδ13C in marine animals
Source: PLoS One. 2018 Jul 18;13(7):e0199680. doi: 10.1371/journal.pone.0199680 (PMC6051570; doi:10.1371/journal.pone.0199680)
Supplement: S1 File — (DOCX) [file pone.0199680.s001.docx]

**S1 File: Appendix**

**Short-term tissue decomposition alters stable isotope values and C:N ratio, but does not change relationships between lipid content, C:N ratio, and Δδ^13^C in marine animals**

Matthew J. Perkins, Yanny. K.Y. Mak, Lily S.R. Tao, Archer T.L. Wong, Jason K.C. Yau, David M. Baker, Kenneth M.Y. Leung

**Table of Contents**

**Supplementary information for the methods………………………………….…….P.2-3**

**Table A. Linear relationships between tissue lipid content (% lipid), C:N ratio and the change in δ^13^C (Δδ^13^C) following removal of lipids………………………………….P.4**

**Supplementary information for the results…………………………………….…….P.5-6**

**Introduction of Tables B-G……………………………………………………………P.7**

**Table B. Minimum adequate model, per species, for δ^15^N…………………………..P.8**

**Table C. Minimum adequate model, per species, for δ^13^C -L……………………….P.9**

**Table D. Minimum adequate model, per species, for δ^13^C +L………………………P.9**

**Table E.** **Minimum adequate model, per species, for %N…………………….……..P.10**

**Table F. Minimum adequate model, per species, for %C…………………….……...P.11**

**Table G. Minimum adequate model, per species, for C:N ratio……………………..P.12**

**Fig A. Changes in tissue measures per individual in Experiment 1…….................P.13-14**

**Table H. Parameter estimates for a LMM testing…………………………………….P.15**

**Supplementary Information for the Methods**

***Experiment 1* - *Q1*. How does tissue decomposition affect *δ*^15^N, *δ*^13^C, %N, %C and C:N ratio?**

**Sample Preparation**

Individuals of two fish species (grouper *Cephalopholis boenak*; rabbitfish *Siganus canaliculatus*), two crustacean species (mantis shrimp *Miyakea nepa*; crab *Portunus sanguinoletus*) and two mollusc species (gastropod *Babylonia areolata*; bivalve *Paphia amabilis*) were assigned randomly to air and ice treatments (*n* = 4 per species per treatment). The air treatment provided a control to quantify the extent of natural tissue decomposition, while the ice treatment evaluated the effectiveness of using an ice covering to reduce any decomposition effects. Repeated tissue sampling per individual was undertaken at (as close as possible) 0, 30, 60, 90 and 120 h. Repeated measures within individuals were used because the results of a pilot experiment suggested that large between-individual variations in derived response variables might mask decomposition effects. Fish and mantis shrimp remained whole, with dorsal muscle and abdominal muscle sampled, respectively. Due to difficulties of separating tissues because of decomposition / drying over time, crabs and molluscs were completely dissected fresh (Time = 0), with claw and leg muscle (crab), foot muscle (gastropod), or foot and adductor muscle (bivalve) pooled per individual on a petri dish from which subsequent samples were taken over time. Both air and ice treatments provided aerobic conditions. ‘Air’ treatment samples were kept in open trays within the laboratory (temperature (±SD) = 20.91°C ± 0.81°C, *n* = 10; humidity = 69.82% ± 3.22%, *n* = 10), while ‘ice’ treatment samples were kept in open freezer bags placed on ice with an additional shallow covering of ice (with tops of bags emergent) within a closed freezer box (temperature = 00.08°C ± 0.86°C, *n* = 10; humidity = 50.65% ± 2.10%, *n* = 10). Ice was replaced every 12 h (before approximately 50% melting) while freezer bags ensured ice and melt water did not contact the samples. Collected samples were immediately freeze dried for >48 h, homogenized and stored under dry conditions prior to stable isotope analysis.

**Stable Isotope Analysis**

For all samples, 1.00mg ± 0.10mg dried tissue was enclosed in tin capsules. Stable Isotope Analysis (SIA) was conducted in the Stable Isotope Ratio Mass Spectrometry Laboratory (SIRMS) at the University of Hong Kong. Samples were analysed for *δ*^15^N, *δ*^13^C, %N and %C in a Eurovector EA3028 elemental analyzer (Isomass Scientific Inc., Alberta, Canada), coupled with a Nu Perspective isotope ratio mass spectrometer (Nu Instruments Ltd, Wrexham, United Kingdom). Stable isotope ratios are reported in delta (*δ*) notation where *δ*^15^N and *δ*^13^C = [(R_sample_/ R_standard_) - 1] x 1000, where R is ^15^N/^14^N or ^13^C/^12^C. Isotope ratios are expressed in per mil (‰) relative to the ratio of international reference standards (R_standard_) which are atmospheric nitrogen and Vienna PeeDee Belemnite (VPDB) for nitrogen and carbon, respectively. Measures of standards placed throughout samples exhibited acceptable instrument reproducibility (standard deviations) of < 0.2‰ for *δ*^15^N, < 0.1‰ for *δ*^13^C, < 0.3% for %N, and < 1.4% for %C, using a certified acetanilide standard (Indiana University) and an in-house fish standard (grouper *Epinephelus awoara*). For each sample, C:N ratio was subsequently calculated by dividing %C by %N.

**Table A**. **Linear relationships between tissue lipid content (% lipid), C:N ratio and the change in *δ*^13^C (*Δδ*^13^C) following removal of lipids.** Relationships for fish and crustacean tissues were significant, while those for molluscs were not. In experiment 1, tissue measures of *δ*^13^C for fish and crustaceans were corrected *a posteriori* using equation 3 and 6, respectively. In experiment 2, we assessed the impact of tissue decomposition upon three methodological procedures for estimating *δ*^13^C in fish tissues; for the *a posteriori* mathematical correction method, we used equation 3 below.

|  | Equation | Sample | Significance | Variance |
| --- | --- | --- | --- | --- |
|  |  | size | (*P* values) | Explained |
|  |  | (*n*) |  | (*R^2^*) |
|  |  |  |  |  |
| Fish |  |  |  |  |
| *Δδ*^13^C = 0.09 + (0.12 x % lipid) | 1 | 20 | **<0.001** | 0.89 |
| % lipid = -24.70 + (9.19 x C:N) | 2 | 20 | **<0.001** | 0.92 |
| *Δδ*^13^C = -2.98 + (1.11 x C:N) | 3 | 20 | **<0.001** | 0.90 |
|  |  |  |  |  |
| Crustacea |  |  |  |  |
| *Δδ*^13^C = 0.06 + (0.17 x % lipid) | 4 | 6 | **<0.01** | 0.90 |
| % lipid = -29.47 + (9.97 x C:N) | 5 | 6 | **<0.05** | 0.78 |
| *Δδ*^13^C = -5.71 + (1.92 x C:N) | 6 | 6 | **<0.01** | 0.90 |
|  |  |  |  |  |
| Mollusca |  |  |  |  |
| *Δδ*^13^C = 0.81 + (-0.002 x % lipid) |  | 10 | 0.99 |  |
| % lipid = 2.00 + (0.31 x C:N) |  | 10 | 0.76 |  |
| *Δδ*^13^C = 0.48 + (0.09 x C:N) |  | 10 | 0.73 |  |

The above equations were derived using a separate set of samples from those used in experiments 1 and 2. Samples were dissected (as per the methods for experiment 1, above), lipid extracted and quantified (as per the methods for experiment 2 in main article), and then underwent stable isotope analysis (as per the methods for experiment 1, above).

**Supplementary Information for the Results**

***Experiment 1* - *Q1*. How does tissue decomposition affect *δ*^15^N, *δ*^13^C, %N, %C and C:N ratio?**

Here we provide further detail on non-significant results and those with little relevance to our study, which were generated in the course of our analyses.

***δ*^15^N**

There was no consistent change in *δ*^15^N over time for mollusc tissues kept in either air or ice (Fig. 1, main article), though variability in *δ*^15^N within an individual ranged from 0.3‰ to 1.4‰ (mean variability = 0.7‰ ± 0.3‰ SD) across the 120 h (Fig. S1), suggesting within-tissue differences in *δ*^15^N were greater than any effects of tissue decomposition.

***δ*^13^C**

We observed no consistent change in *δ*^13^C over 120 h for either Grouper (for either *δ*^13^C -L or *δ*^13^C +L) or Gastropod (*δ*^13^C +L only) tissues in either air or ice (Fig. 1, main article).

**%N**

There was no consistent change in %N over time for mollusc tissues kept in either air or ice (Fig. 1, main article), though variability in %N within an individual ranged from 0.82% to 3.33% (mean variability = 1.86% ± 0.72% SD) across 120 h (Fig. S1), suggesting within-tissue differences in %N were likely greater than any effects from tissue decomposition.

**%C**

For Crab, there was no change in %C of tissues in either air or ice (Fig. 1, main article). For both mollusc analyses, we observed only significant main effects of *treatment* on %C, suggesting an unimportant artefact of a random sampling regime whereby air treatments had lower mean %C than ice treatments (mean ± SD: Gastropod air = 42.41 ± 1.45 vs ice = 43.61 ± 1.02; Bivalve air = 41.97 ± 0.95 vs ice = 42.99 ± 1.17).

**C:N ratio**

For Rabbitfish, it was noted that tissues kept in air showed increases in C:N ratios and those in ice did not change (i.e. the same pattern as Grouper, Mantis Shrimp and Crab), if the single highest C:N ratio at T_0_ for one air individual (Fig. S1) was removed. However, we have no justification for its removal, so instead only an unimportant main effect of *treatment* was found, with samples kept in air on average having higher C:N ratios than those kept in ice (mean ± SD: air = 3.37 ± 0.19 vs ice = 3.23 ± 0.13; Fig. 1). For molluscs, there was no consistent change in C:N ratios over 120 h for tissues kept in either air or ice (Fig. 1, main article), though notable within-individual variability in C:N ratios ranging from 0.15 to 1.21 (mean variability = 0.56 ± 0.33 SD) across the 120 h period suggested within-tissue differences were greater than any effects of tissue decomposition (Fig. S1).

**Introduction of Tables B – G**

**In experiment 1, we used linear mixed models (LMM) to test if *time*, *treatment* (air or ice) or their interaction affected a suite of tissue measures.** Separate analyses were conducted per tissue measure per species. For each analysis, we included the random effect *individual* to account for non-independence of repeated measures per individual. Significance of an interaction effect was determined using analysis of deviance between models with and without the interaction included. Interactions were dropped when they were not significant, and subsequently main effects were similarly tested by analysis of deviance between models with and without each term, until all non-significant terms were eliminated and a minimum adequate model was identified. When a significant interaction was determined, slopes for either air or ice treatments were determined as significant if estimates for 95% CI did not pass through zero. Below we report minimum adequate models with significant model terms. Significant model terms and their parameter estimates are highlighted in bold. For clarity, below each model output, we also report the slope estimates for air and ice treatments, and the magnitude of change after 120 h when significant.

**Table B**. Minimum adequate model, per species, for δ^15^N.

|  |  |  |  |  |  |
| --- | --- | --- | --- | --- | --- |
|  | Estimate | Std. Error | 2.50% CI | 97.50% CI | Change: 0h to 120h |
| *Grouper* |  |  |  |  |  |
|  |  |  |  |  |  |
| Intercept | 15.9362 | 0.2387 | 15.4683 | 16.4040 |  |
| Time | 0.0061 | 0.0009 | 0.0044 | 0.0078 |  |
| Treatment | -0.2686 | 0.3365 | -0.9281 | 0.3909 |  |
| **Time x Treatment** | **-0.0058** | **0.0012** | **-0.0081** | **-0.0035** |  |
|  |  |  |  |  |  |
| **Slope Air** | **0.0061** | **0.0009** | **0.0044** | **0.0078** | **0.7** |
| Slope Ice | 0.0003 | 0.0008 | -0.0013 | 0.0018 |  |
|  |  |  |  |  |  |
| *Rabbitfish* |  |  |  |  |  |
|  |  |  |  |  |  |
| Intercept | 12.0458 | 0.1758 | 11.7012 | 12.3903 |  |
| Time | 0.0108 | 0.0012 | 0.0084 | 0.0132 |  |
| Treatment | -0.1627 | 0.2486 | -0.6500 | 0.3245 |  |
| **Time x Treatment** | **-0.0093** | **0.0018** | **-0.0127** | **-0.0058** |  |
|  |  |  |  |  |  |
| **Slope Air** | **0.0108** | **0.0012** | **0.0084** | **0.0132** | **1.3** |
| Slope Ice | 0.0015 | 0.0012 | -0.0009 | 0.0040 |  |
|  |  |  |  |  |  |
| *Mantis Shrimp* |  |  |  |  |  |
|  |  |  |  |  |  |
| Intercept | 12.5225 | 0.1970 | 12.1364 | 12.9087 |  |
| Time | 0.0082 | 0.0011 | 0.0059 | 0.0104 |  |
| Treatment | -0.8019 | 0.2786 | -1.3481 | -0.2558 |  |
| **Time x Treatment** | **-0.0044** | **0.0016** | **-0.0076** | **-0.0013** |  |
|  |  |  |  |  |  |
| **Slope Air** | **0.0082** | **0.0011** | **0.0059** | **0.0104** | **1.0** |
| **Slope Ice** | **0.0037** | **0.0011** | **0.0015** | **0.0059** | **0.4** |
|  |  |  |  |  |  |
| *Crab* |  |  |  |  |  |
|  |  |  |  |  |  |
| Intercept | 12.4004 | 0.1760 | 12.0554 | 12.7454 |  |
| Time | 0.0052 | 0.0012 | 0.0028 | 0.0076 |  |
| Treatment | -0.7055 | 0.2490 | -1.1934 | -0.2175 |  |
| **Time x Treatment** | **-0.0052** | **0.0017** | **-0.0086** | **-0.0017** |  |
|  |  |  |  |  |  |
| **Slope Air** | **0.0052** | **0.0012** | **0.0028** | **0.0076** | **0.6** |
| Slope Ice | 0.0000 | 0.2381 | -0.4666 | 0.4667 |  |

**Table C.** Minimum adequate model, per species, for δ^13^C -L.

|  |  |  |  |  |  |
| --- | --- | --- | --- | --- | --- |
|  | Estimate | Std. Error | 2.50% CI | 97.50% CI | Change: 0h to 120h |
| *Rabbitfish* |  |  |  |  |  |
|  |  |  |  |  |  |
| Intercept | -17.8000 | 0.1962 | -18.1846 | -17.4154 |  |
| **Time** | **0.0017** | **0.0005** | **0.0007** | **0.0028** | **0.2** |
|  |  |  |  |  |  |
| *Mantis Shrimp* |  |  |  |  |  |
|  |  |  |  |  |  |
| Intercept | -14.7704 | 0.1257 | -15.0169 | -14.5240 |  |
| Time | 0.0032 | 0.0011 | 0.0011 | 0.0054 |  |
| Treatment | 0.3248 | 0.1778 | -0.0238 | 0.6733 |  |
| **Time x Treatment** | **-0.0049** | **0.0015** | **-0.0080** | **-0.0019** |  |
|  |  |  |  |  |  |
| **Slope Air** | **0.0032** | **0.0011** | **0.0011** | **0.0054** | **0.4** |
| Slope Ice | -0.0017 | 0.0011 | -0.0038 | 0.0004 |  |
|  |  |  |  |  |  |
| *Crab* |  |  |  |  |  |
|  |  |  |  |  |  |
| Intercept | -15.8100 | 0.1115 | -16.0285 | -15.5915 |  |
| Time | 0.0023 | 0.0007 | 0.0009 | 0.0036 |  |
| Treatment | -0.3364 | 0.1576 | -0.6453 | -0.0275 |  |
| **Time x Treatment** | **-0.0034** | **0.0010** | **-0.0053** | **-0.0014** |  |
|  |  |  |  |  |  |
| **Slope Air** | **0.0023** | **0.0007** | **0.0009** | **0.0036** | **0.3** |
| Slope Ice | -0.0011 | 0.0007 | -0.0025 | 0.0003 |  |

**Table D.** Minimum adequate model, per species, for δ^13^C +L.

|  |  |  |  |  |  |
| --- | --- | --- | --- | --- | --- |
|  | Estimate | Std. Error | 2.50% CI | 97.50% CI | Change: 0h to 120h |
| *Rabbitfish* |  |  |  |  |  |
|  |  |  |  |  |  |
| Intercept | -17.6500 | 0.1947 | -18.0316 | -17.2684 |  |
| **Time** | **0.0017** | **0.0005** | **0.0007** | **0.0027** | **0.2** |
|  |  |  |  |  |  |
| *Mantis Shrimp* |  |  |  |  |  |
|  |  |  |  |  |  |
| Intercept | -15.1897 | 0.1311 | -15.4468 | -14.9327 |  |
| Time | -0.0088 | 0.0013 | -0.0114 | -0.0062 |  |
| Treatment | 0.4150 | 0.1855 | 0.0515 | 0.7784 |  |
| **Time x Treatment** | **0.0058** | **0.0019** | **0.0021** | **0.0095** |  |
|  |  |  |  |  |  |
| **Slope Air** | **-0.0088** | **0.0013** | **-0.0114** | **-0.0062** | **-1.1** |
| **Slope Ice** | **-0.0031** | **0.0013** | **-0.0057** | **-0.0004** | **-0.4** |
|  |  |  |  |  |  |
| *Bivalve* |  |  |  |  |  |
|  |  |  |  |  |  |
| Intercept | -18.8900 | 0.1038 | -19.0934 | -18.6866 |  |
| **Time** | **-0.0028** | **0.0006** | **-0.0040** | **-0.0016** | **-0.3** |

**Table E.** Minimum adequate model, per species, for %N.

|  |  |  |  |  |  |
| --- | --- | --- | --- | --- | --- |
|  | Estimate | Std. Error | 2.50% CI | 97.50% CI | Change: 0h to 120h |
| *Grouper* |  |  |  |  |  |
|  |  |  |  |  |  |
| Intercept | 14.1943 | 0.2281 | 13.7472 | 14.6414 |  |
| Time | -0.0166 | 0.0030 | -0.0225 | -0.0107 |  |
| Treatment | -0.3567 | 0.3103 | -0.9650 | 0.2515 |  |
| **Time x Treatment** | **0.0184** | **0.0041** | **0.0104** | **0.0265** |  |
|  |  |  |  |  |  |
| **Slope Air** | **-0.0166** | **0.0030** | **-0.0225** | **-0.0107** | **-1.99** |
| Slope Ice | 0.0018 | 0.0028 | -0.0037 | 0.0074 |  |
|  |  |  |  |  |  |
| *Rabbitfish* |  |  |  |  |  |
|  |  |  |  |  |  |
| Intercept | 13.8807 | 0.2575 | 13.3759 | 14.3855 |  |
| Time | -0.0212 | 0.0032 | -0.0275 | -0.0148 |  |
| Treatment | -0.1164 | 0.3642 | -0.8303 | 0.5974 |  |
| **Time x Treatment** | **0.0265** | **0.0046** | **0.0175** | **0.0354** |  |
|  |  |  |  |  |  |
| **Slope Air** | **-0.0212** | **0.0032** | **-0.0275** | **-0.0148** | **-2.54** |
| Slope Ice | 0.0053 | 0.0032 | -0.0010 | 0.0117 |  |
|  |  |  |  |  |  |
| *Mantis Shrimp* |  |  |  |  |  |
|  |  |  |  |  |  |
| Intercept | 12.6822 | 0.2985 | 12.0971 | 13.2673 |  |
| Time | -0.0286 | 0.0029 | -0.0343 | -0.0229 |  |
| Treatment | 0.2192 | 0.4222 | -0.6082 | 1.0467 |  |
| **Time x Treatment** | **0.0284** | **0.0041** | **0.0204** | **0.0364** |  |
|  |  |  |  |  |  |
| **Slope Air** | **-0.0286** | **0.0029** | **-0.0343** | **-0.0229** | **-3.43** |
| Slope Ice | -0.0002 | 0.0029 | -0.0059 | 0.0055 |  |
|  |  |  |  |  |  |
| *Crab* |  |  |  |  |  |
|  |  |  |  |  |  |
| Intercept | 13.0721 | 0.1123 | 12.8520 | 13.2923 |  |
| Time | -0.0039 | 0.0015 | -0.0069 | -0.0009 |  |
| Treatment | 0.1649 | 0.1588 | -0.1464 | 0.4762 |  |
| **Time x Treatment** | **0.0060** | **0.0022** | **0.0018** | **0.0103** |  |
|  |  |  |  |  |  |
| **Slope Air** | **-0.0039** | **0.0015** | **-0.0069** | **-0.0009** | **-0.47** |
| Slope Ice | 0.0022 | 0.0015 | -0.0009 | 0.0052 |  |

**Table F.** Minimum adequate model, per species, for %C.

|  | Estimate | Std. Error | 2.50% CI | 97.50% CI | Change: 0h to 120h |
| --- | --- | --- | --- | --- | --- |
| *Grouper* |  |  |  |  |  |
|  |  |  |  |  |  |
| Intercept | 45.4812 | 0.6695 | 44.1690 | 46.7935 |  |
| Time | -0.0377 | 0.0088 | -0.0550 | -0.0205 |  |
| Treatment | -0.7278 | 0.9108 | -2.5131 | 1.0574 |  |
| **Time x Treatment** | **0.0436** | **0.0121** | **0.0199** | **0.0673** |  |
|  |  |  |  |  |  |
| **Slope Air** | **-0.0377** | **0.0088** | **-0.0550** | **-0.0205** | **-4.53** |
| Slope Ice | 0.0058 | 0.0083 | -0.0105 | 0.0221 |  |
|  |  |  |  |  |  |
| *Rabbitfish* |  |  |  |  |  |
|  |  |  |  |  |  |
| Intercept | 46.5296 | 0.4403 | 45.6667 | 47.3926 |  |
| Time | -0.0691 | 0.0059 | -0.0806 | -0.0575 |  |
| Treatment | -1.2721 | 0.6226 | -2.4925 | -0.0517 |  |
| **Time x Treatment** | **0.0723** | **0.0083** | **0.0560** | **0.0887** |  |
|  |  |  |  |  |  |
| **Slope Air** | **-0.0691** | **0.0059** | **-0.0806** | **-0.0575** | **-8.29** |
| Slope Ice | 0.0032 | 0.0059 | -0.0083 | 0.0148 |  |
|  |  |  |  |  |  |
| *Mantis Shrimp* |  |  |  |  |  |
|  |  |  |  |  |  |
| Intercept | 40.9004 | 0.5431 | 39.8359 | 41.9650 |  |
| Time | -0.0383 | 0.0053 | -0.0486 | -0.0280 |  |
| Treatment | -0.3777 | 0.7681 | -1.8833 | 1.1278 |  |
| **Time x Treatment** | **0.0470** | **0.0074** | **0.0324** | **0.0616** |  |
|  |  |  |  |  |  |
| **Slope Air** | **-0.0383** | **0.0053** | **-0.0486** | **-0.0280** | **-4.60** |
| Slope Ice | 0.0087 | 0.0053 | -0.0016 | 0.0190 |  |

**Table G.** Minimum adequate model, per species, for C:N ratio.

|  |  |  |  |  |  |
| --- | --- | --- | --- | --- | --- |
|  | Estimate | Std. Error | 2.50% CI | 97.50% CI | Change: 0h to 120h |
| *Grouper* |  |  |  |  |  |
|  |  |  |  |  |  |
| Intercept | 3.2004 | 0.0298 | 3.1420 | 3.2588 |  |
| Time | 0.0013 | 0.0004 | 0.0006 | 0.0021 |  |
| Treatment | 0.0348 | 0.0405 | -0.0447 | 0.1142 |  |
| **Time x Treatment** | **-0.0013** | **0.0005** | **-0.0024** | **-0.0003** |  |
|  |  |  |  |  |  |
| **Slope Air** | **0.0013** | **0.0004** | **0.0006** | **0.0021** | **0.16** |
| Slope Ice | 0.0000 | 0.0004 | -0.0007 | 0.0007 |  |
|  |  |  |  |  |  |
| *Mantis Shrimp* |  |  |  |  |  |
|  |  |  |  |  |  |
| Intercept | 3.1896 | 0.0722 | 3.0480 | 3.3312 |  |
| Time | 0.0063 | 0.0008 | 0.0047 | 0.0079 |  |
| Treatment | -0.0469 | 0.1022 | -0.2472 | 0.1533 |  |
| **Time x Treatment** | **-0.0056** | **0.0011** | **-0.0078** | **-0.0033** |  |
|  |  |  |  |  |  |
| **Slope Air** | **0.0063** | **0.0008** | **0.0047** | **0.0079** | **0.75** |
| Slope Ice | 0.0007 | 0.0008 | -0.0009 | 0.0023 |  |
|  |  |  |  |  |  |
| *Crab* |  |  |  |  |  |
|  |  |  |  |  |  |
| Intercept | 3.1770 | 0.0190 | 3.1398 | 3.2141 |  |
| Time | 0.0012 | 0.0002 | 0.0008 | 0.0016 |  |
| Treatment | -0.0451 | 0.0268 | -0.0976 | 0.0074 |  |
| **Time x Treatment** | **-0.0011** | **0.0003** | **-0.0017** | **-0.0005** |  |
|  |  |  |  |  |  |
| **Slope Air** | **0.0012** | **0.0002** | **0.0008** | **0.0016** | **0.14** |
| Slope Ice | 0.0001 | 0.0002 | -0.0004 | 0.0005 |  |

**
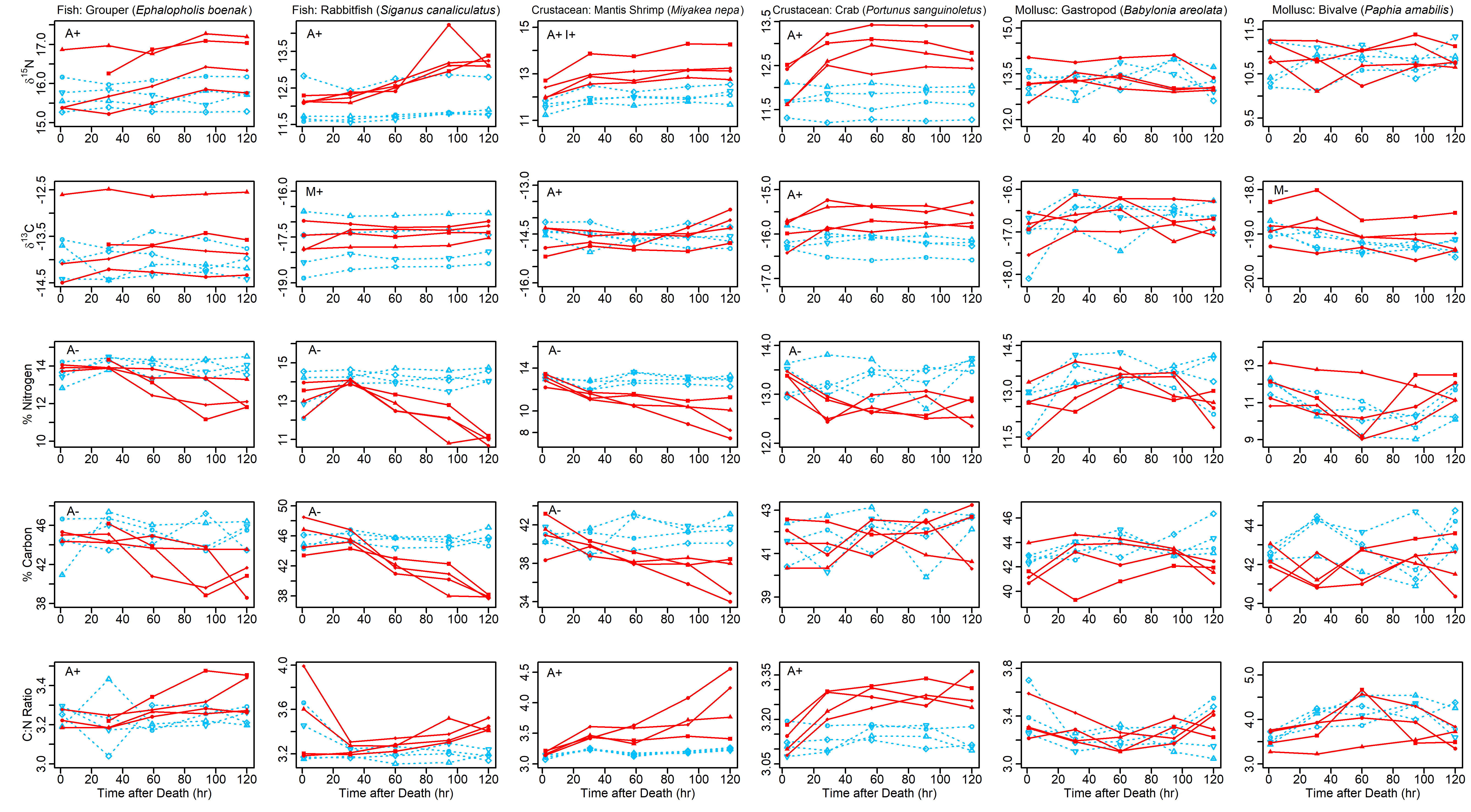
**

**Figure A.** **Changes in tissue measures per individual in Experiment 1.** Change in *δ^15^N, δ^13^C, %N, %C* and *C:N ratio* over 120 h for fish, crustacean and mollusc tissues kept in air (solid red lines) or ice (dotted blue lines). Per species per variable, each line represents an individual (*n* = 4 air, 4 ice). Symbol type identifies five repeated measures taken per individual over time per variable, and individual across variables. For δ^13^C, fish and crustaceans use δ^13^C -L, while molluscs use δ^13^C +L. Letters denote a significant interaction whereby effects of time upon response variable differ between air (A) and in ice (I) treatments, or a significant main effect of time on response variable that does not differ between air and ice (M). + / - indicates the direction of effect.

**Results**

***Experiment 2* - *Q2.* Does tissue decomposition affect tissue lipid content or relationships between (i) lipid content and *Δδ*^13^C, (ii) C:N ratio and lipid content, and (iii) C:N ratio and *Δδ*^13^C?**

**Table H.** **Parameter estimates for a LMM testing: lipid content ~ Time × Treatment + (1|individual).** The interaction, and subsequent main effects, were not significant, indicating lipid content was not affected by tissue decomposition in either air or ice.

|  | Estimate | Std. Error | 2.50 % CI | 97.50 % CI |
| --- | --- | --- | --- | --- |
|  |  |  |  |  |
| Intercept | 16.7711 | 2.6934 | 11.4921 | 22.0501 |
| Time | 0.0065 | 0.0146 | -0.0221 | 0.0351 |
| Treatment | -2.8803 | 3.8090 | -10.3459 | 4.5853 |
| Time x Treatment | -0.0389 | 0.0206 | -0.0793 | 0.0014 |
|  |  |  |  |  |
